# Gender-specific contributing risk factors and outcome of female cryptococcal meningoencephalitis patients

**DOI:** 10.1186/s12879-016-1363-z

**Published:** 2016-01-22

**Authors:** Hui Zheng, Mingyue Li, Dongmei Wang, Jia ling Yang, Qiong Chen, Xiaomei Zhang, Yang Man, Junying Lao, Ningfen Chen, Suyue Pan

**Affiliations:** 1 Department of Neurology, Nanfang Hospital, Southern Medical University, No.1838 North Guangzhou Avenue, Guangzhou City, Guangdong Province People’s Republic of China; 2 Medical Records Room, Nanfang Hospital, Southern Medical University, Guangzhou City, Guangdong Province People’s Republic of China

**Keywords:** Cryptococcal meningoencephalitis, Female, Gender-specific, Retrospective research, Contributing risk factor, Survival length

## Abstract

**Background:**

Although male predominance was documented in previous studies on cryptococcal meningoencephalitis (CM), there has been no statistical study about female CM patients despite recently noticeable increase in female prevalence. In the current study, we aimed to investigate the independent gender-specific contributing risk factors for onset of CM and factors related to survival time in female patients by chosen statistical tools.

**Methods:**

There have been 108 patients diagnosed with CM from July 1, 1998 to June 30, 2013 in Nanfang Hospital that were included in our study. This 15-year retrospective study compared demographic and clinical features of 31 female patients with 77 males. Multivariate analysis was performed for detection of the contributors to the onset of CM in female patients. The independent variables for multivariate analysis were selected according to statistical significance in univariate analysis. Furthermore, Cox regression model was used to evaluate the factors related to survival length.

**Results:**

Use of corticosteroids or other immunosuppressants (32.3 % versus 11.7 %; *p* = 0.011) and history of systemic lupus erythematosus (SLE) and other autoimmune diseases (29 % versus 3.9 %; *p* < 0.001) were more common in females, but only the history of SLE or other autoimmune diseases was significant (OR 10.59, 95 % CI 1.49-74.77, *p* = 0.02) by multivariate analysis. The ratio of cerebrospinal fluid (CSF) glucose-to-blood glucose was related to the survival time (*p* = 0.03, 95 % CI 0-0.71).

**Conclusions:**

The results showed that the history of SLE or other autoimmune diseases rather than chronic use of corticosteroids and/or immunosuppressants was the independent gender-specific contributing risk factor in female CM patients. Therefore, more attention should be made to the prevention of infection from the genus *Cryptococcus spp.* in female patients with SLE or other autoimmune diseases. In addition, decreased ratio of CSF glucose-to-blood glucose before antifungal therapy predicted the worse prognosis.

## Background

The genus *Cryptococcus spp.* is an encapsulated yeast-like fungus which causes life-threatening diseases and can infect some important organs in the human body [1, 2]. Its infection of the central nervous system (CNS)—cryptococcal meningoencephalitis (CM) - is one of the most common fungal infection causing morbidity and mortality worldwide [3, 4].

Many diseases have a different frequency and severity between female and male [5, 6], a tendency featured in CM. Male predominance was apparent in most of the previous CM studies in different countries [1, 7–10], but recently the noticeable increase in female prevalence was seen, as suggested by the decreased male-to-female ratio [10, 11]. Until now, several reports have called attention to the female CM patients [12–16], but no statistical study of these patients was found.

In the current study, by observing the gender differences of the epidemiological characteristics, clinical features and outcome in patients diagnosed with CM in Nanfang Hospital in China, we firstly demonstrated the independent gender-specific contributing risk factors for the onset of CM and factors related to survival time in female CM patients by chosen statistical tools.

## Methods

### Data source and study population

In this retrospective observational study, 108 patients diagnosed with CM from Nanfang Hospital were included between July 1, 1998 to June 30, 2013 (15 years). Their medical records were reviewed to extract gender-specific demographic and clinical features, including potential risk factors, symptoms, cerebrospinal fluid (CSF) analysis, electroencephalograph (EEG) and magnetic resonance imaging (MRI) results. Furthermore, the probable contributing risk factors and survival time of female CM patients were analyzed. All patients or their family members signed a written consent in accordance with the ethical committee standards during their hospital stay or outpatient follow-up and the written approval for this study was obtained from the ethics committee of the Nanfang Hospital.

### Study criteria

CM was defined by either of the following criteria: (1) isolation of the genus *Cryptococcus spp.* from previous or current cerebrospinal fluid cultures, followed by a positive CSF cryptococcal antibody, positive CSF India ink staining, or positive CSF Aley new blue dye staining and clinical features of meningoencephalitis; or (2) isolation of the genus *Cryptococcus spp.* in blood culture with clinical presentations of meningoencephalitis and typical CSF features [17].

Patients were regarded as “with potential risk factors” when they have HIV infection, malignancies, cirrhosis, organ transplantation, end-stage renal failure autoimmune disorder, diabetes mellitus, idiopathic CD4 T-cell lymphopenia, sarcoidosis, chronic usage of corticosteroids or other immunosuppressive therapy [18], and systemic lupus erythematosus (SLE) or other systemic autoimmune diseases, chronic kidney diseases, pregnant, mycosis infection of other system, drug addiction, etc.

Bubble sign in MRI was diagnosed as follows [19]: lesions frequently seen in the basal ganglia, thalami, and midbrain, hyperintensity on T2-weighted images and fluid attenuated inversion recovery (FLAIR) images without contrast enhancement.

### Therapy

The 108 patients received treatment with an intravenous administration of amphotericin B deoxycholate (AmBd), which was given at 0.1 mg/kg the first day, 0.5 mg/kg the second day and was increased to 1 mg/kg per day from the third day, together with an oral 5-fluorocytosine (5-FC) at 100 mg/kg per day and fluconazole (FCZ) at 400 mg per day, once they were diagnosed with CM. After 4–6 weeks of induction treatment, if the genus *Cryptococcus spp.* was no longer present in at least three sequential CSF examinations by microscopy, we stopped AmBd. We continued to use FCZ and 5-FC for 6–9 more months. The patients with persistent high intracranial pressure received a lumber puncture every 2 or 3 days to relieve their symptoms.

### Data analysis

For comparison of continuous variables, such as age, CSF pressure, CSF protein, CSF glucose level, ratio of CSF glucose-to-blood glucose, hospitalization length and survival time, an independent sample t-test was performed. Chi-square test was used to compare the categorical variable, including bird/ bird dropping contact history, potential risk factors, symptoms, EEGs, MRI results and outcome status between female and male. Multivariate analysis was performed for detection of the contributors to the onset of CM in female patients. The independent variables for multivariate analysis were selected according to statistical significance in univariate analysis. Furthermore, Cox regression model was used to evaluate the factors related to survival length. Statistical significance was defined as *p* < 0.05 and data was analyzed using SPSS 16.0 software (SPSS, Chicago, IL, USA).

## Results

Among the 108 CM patients collected from Nanfang Hospital, 31 of them were female (Mean age33.55 ± 15.65 years old; Range 5–63 years old). Average time interval from illness onset to hospitalization was 47.48 ± 82.42 days. Two cases had clear history of exposure to bird/bird droppings. There were 19 of 31 cases (61.3 %) that had potential risk factors. The most common potential risk factor was chronic use of corticosteroids or other immunosuppressants (10 cases, 32.3 %), and then SLE or other autoimmune diseases (9 cases, 29.0 %), kidney diseases (3 cases, 9.7 %), diabetes mellitus (1 case, 3.2 %), tuberculosis (1 case, 3.23 %), hyperthyroidism (1 case, 3.23 %), pregnant (1 case, 3.23 %), hepatitis B infection (1 case, 3.23 %) and gastric ulcer (1 case, 3.23 %) (Table 1). Compared with male patients, the history of SLE or other antoimmune diseases and the use of corticosteroids and/or other immunosuppressants had more relationship to the onset of CM in female patients.

**Table 1:** Demographic characteristics, contact history and risk factors related to gender in CM patients

	Male (*n* = 77)	Female (*n* = 31)	*P*
Demographic characteristics			
Age (years), mean ± SD	38.95 ± 18.32	33.55 ± 15.65	0.15
Contact history, no. (%)	3 (3.9)	2 (1.4)	0.624
Risk factors			
Use of corticosteroids and/or immunosuppressants, no. (%)	9 (11.7)	10 (32.3)	0.011^*^
History of SLE or other autoimmune diseases, no. (%)	3 (3.9)	9 (29)	<0.001^*^
History of kidney diseases^a^, no. (%)	1 (1.3)	3 (9.7)	0.07
History of hematopathy, no. (%)	2 (2.6)	0 (0)	0.506
History of DM, no. (%)	4 (5.2)	1 (3.2)	0.554
History of TB, no. (%)	5 (6.5)	1 (3.2)	0.444
History of other diseases^b^, no. (%)	2 (2.6)	3 (9.7)	0.145
History of malignant cancer, no. (%)	1 (1.3)	0 (0)	0.713
History of organ transplantation, no. (%)	1 (1.3)	0 (0)	0.713

^*^ *P* < 0.05

^a^Kidney diseases included renal failure 2 cases and RPGN 1 case

^b^Other diseases included hyperthyroidism 1 case, pregnant 1 case and gastric ulcer 1 case

Nonspecific symptoms, such as headache (24 cases, 77.42 %) and fever (19 cases, 61.29 %) were frequently found in female CM patients, while seizures (3 cases, 9.7 %), dizziness (1 case, 3.2 %), ataxia (1 case, 3.2 %) and psychosis (2 cases, 6.5 %) were rare. Although female were more inclined to visit a doctor because of unconsciousness (25.8 % vs. 14.3 %), seizures (9.7 % vs. 6.5 %) and dizziness (3.2 % vs. 1.3 %), there was no statistical difference (Table 2). In addition, there was no difference between male and female in the first CSF results, containing CSF pressure, CSF glucose level, CSF protein, the ratio of CSF glucose-to-blood glucose, EEG examinations, and MRI results (Table 2).

**Table 2:** Clinical features and outcome related gender in CM patients

	Male (*n* = 77)	Female (*n* = 31)	*P*
Symptoms			
Headache, no. (%)	57 (74.0)	24 (77.4)	0.713
Fever, no. (%)	48 (62.3)	19 (61.3)	0.919
Seizure, no. (%)	5 (6.5)	3 (9.7)	0.687
Dizziness, no. (%)	1 (1.3)	1 (3.2)	0.494
Ataxia, no. (%)	3 (3.9)	1 (3.2)	1
Unconsciousness, no. (%)	11 (14.3)	8 (25.8)	0.155
Psychosis, no. (%)	2 (2.6)	2 (6.5)	0.577
CSF, mean ± SD			
Ratio of CSF glucose/Blood glucose, mean ± SD	0.30 ± 0.18	0.28 ± 0.18	0.54
CSF pressure (mmH2O), mean ± SD	266.49 ± 60.13	271.94 ± 49.49	0.66
CSF glucose (mmol/L), mean ± SD	2.08 ± 1.36	1.64 ± 1.01	0.80
CSF protein (g/L), mean ± SD	0.94 ± 1.53	0.86 ± 0.51	0.11
Abnormal EEG^a^, no. (%)	17 (68.0)	10 (76.9)	0.71
Abnormal MRI^b^, no. (%)	25 (75.8)	9 (60.0)	0.31
Outcome			
Hospitalization length (days), mean ± SD	50.71 ± 55.36	53.35 ± 53.93	0.44
Survival time (months), mean ± SD	30.40 ± 45.30	21.22 ± 36.15	0.09
Dead, no. (%)	30 (45.5)	15 (53.6)	0.47

^a^Abnormal EEG included 5 cases had normal background with focal slow wave and 5 had diffused slow wave in the background

^b^Abnormal MRI included Bubble sign 5 cases (2 at the basal ganglion and 3 at subcortex), hydrocephalus 5 cases and 6 cases with abnormal cerebral lesions (6 cases with more than one abnormal features)

There was no difference of hospitalization length (53.35 ± 53.93 days vs. 50.71 ± 55.36 days, *p* = 0.44) and survival time (21.22 ± 36.15 months vs. 30.40 ± 45.30 months, *p* = 0.09) between female and male CM patients. The mortality of female CM patients was slightly higher (15 cases, 53.6 %) than male (30 cases, 45.5 %), but without statistical significance (Table 2).

On our univariate analysis, use of corticosteroids and/or immunosuppressants (32.3 % versus 11.7 %; *p* = 0.011) and history of SLE or other autoimmune diseases (29 % versus 3.9 %; *p* < 0.001) were significantly higher in female CM patients. However, on multivariate analysis, we found history of SLE or other autoimmune diseases was significant between genders (odds ratio (OR) 10.59, 95 % confidence interval (CI) 1.49-74.77, *p* = 0.02), while the use of corticosteroids and/or immunosuppressants was not (OR 0.94, 95 % CI 0.18-4.86, *p* = 0.95) (Table 3).

**Table 3:** Multivariate conditional logistic analysis of independent risk factors for onset of CM in female patients (*n* = 31)

	Univariate analysis			Multivariate analysis		
	OR	95 % CI	*P*	OR	95 % CI	*P*
Use of corticosteroids and/or immunosuppressants	2.23	1.27–3.93	0.011^*^	0.94	0.18–4.86	0.95
History of SLE or other autoimmune diseases	3.27	2.00–5.35	<0.001^*^	10.59	1.49–74.77	0.02^*^

^*^ *P* < 0.05

Cox regression analysis of female CM patients demonstrated that the decreased ratio of CSF glucose-to-blood glucose was significantly related to survival time in female patients (*p* = 0.03, 95 % CI 0-0.71) while decreased consciousness (*p* = 0.18, 95 % CI 0.07-1.65) and CSF pressure (*p* = 0.41, 95 % CI 0.99-1.00) were not (Table 4).

**Table 4:** Cox regression analysis of risk factors for survival length of female cryptococcal meningoencephalitis patients

Risk factors	P	OR	95 % CI
Decreased consciousness level	0.18	0.345	0.07–1.65
CSF pressure	0.41	0.996	0.99–1.00
The ratio of CSF glucose/ blood glucese	0.03^*^	0.018	0.00–0.71

^*^ *P* < 0.05

## Discussion

The genus *Cryptococcus spp.* can infect almost all organs in the human body, but the site of serious consequence is the central nervous system [2, 3]. Despite the improvements in diagnosis and management, lack of specific symptoms, long hospitalization and high mortality still make CM a significant challenge to clinical works [20, 21]. In this study, we compared the demographic and clinical features of 31 female and 77 male CM patients, and attempted to analyze the independent risk factor for onset of CM in female and the factors related to their survival time.

The results of this study revealed several significant features between female and male CM patients. In female CM patients, use of corticosteroids and/or immunosuppressants and the history of SLE or other autoimmune diseases were more common, but history of SLE or other autoimmune diseases was the only clinical risk factor independently contributed to the onset. Similar to our results from a previous study involving the whole population [9], the ratio of CSF glucose-to-blood glucose was the only factor related to the survival length of female CM patients.

Kwon et al. [18] suggested several risk factors for CM including HIV infection, malignancies, cirrhosis, organ transplantation, end-stage renal failure autoimmune disorder, diabetes mellitus, idiopathic CD4 T-cell lymphopenia, sarcoidosis, chronic usage of corticosteroids or other immunosuppressive therapy. While Yuchong et al. [3] found that tuberculosis and liver diseases were more common potential risk factors in China. Additionally, Guo et al. [22] suggested contact with birds/bird droppings or saprophytes before illness onset in children. In our study, the gender-specific risk factors in female CM were use of corticosteroids and/or immunosuppressants and the history of SLE or other autoimmune diseases. This can probably be explained due to the marked gender predilections with women more commonly afflicted than men.

Surprisingly, the history of SLE or other autoimmune diseases rather than the chronic use of corticosteroids and/or immunosuppressants were the independent gender-specific contributing risk factor to onset of CM in female patients. Previous studies reported that efficient control of cryptococcal infection requires a delicate balance of both Th1- and Th2- type responses [23, 24]. It probably because that T cells play a major role in SLE by amplifying the autoimmune response leading to a misbalance of the downstream cytokines [25], while the balance remains in the inhibition of T-cell proliferation and activation caused by use of corticosteroids and immunosuppressants. This result demonstrated that once diagnosed with SLE or other autoimmune diseases, the female patients should be given prevention of the cryptococcal infection, independent of chronic use of corticosteroids or other immunosuppressants.

Decreased consciousness level, a lower CSF opening pressure and the decreased ratio of CSF glucose-to-blood glucose were reported contributed to CM prognosis [26]. In our study, decreased ratio of CSF glucose-to-blood glucose was the only factor related to the survival length of female CM patients. This result illustrated that the female CM patients who had decreased ratio of CSF glucose-to-blood glucose in the first CSF analysis should monitored closely during the therapy process, for they may have longer hospitalization time and higher mortality.

This study had limitations inherent to all retrospective studies, such as inconsistencies in patients care. Another limitation results from lost contact with cases during the follow-up process, particularly of cases in rural areas where it is difficult to reach by mail, phone or car. One of the strengths of our study is firstly demonstrating the gender-specific risk factor for the onset of CM in female patients as well as our use of Cox regression analysis to assess the factors related to survival time in them.

## Conclusions

The genus *Cryptococcus spp.* infection of CNS increased in female patients in recent years. Use of corticosteroids or other immunosuppressants and history of SLE and other autoimmune diseases were more common in females than males. However, the history of SLE or other autoimmune diseases rather than chronic use of corticosteroids and/or immunosuppressants was the independent gender-specific contributing risk factor to onset of CM in female patients. Decreased ratio of CSF glucose-to-blood glucose in the CSF analysis before antifungal therapy predicted the worse prognosis. So once diagnosed with SLE or other autoimmune diseases, the female patients should be given prevention of the cryptococcal infection, independent of chronic use of corticosteroids or other immunosuppressants. Furthermore the female patients who had decreased ratio of CSF glucose-to-blood glucose in the first CSF analysis should be monitored closely during the therapy process.

## Abbreviations

5-FC: 5-fluorocytosine
AmBd: amphotericin B
CI: confidence interval
CM: cryptococcal meningoencephalitis
CNS: central nervous system
CSF: cerebrospinal fluid
EEG: electroencephalograph
FCZ: fluconazole
FLAIR: fluid attenuated inversion recovery
MRI: magnetic resonance imaging
OR: odds ratio
SD: standard deviation
SLE: systemic lupus erythematosus
